# Intranasal Dexmedetomidine With Intravenous Midazolam: A Safe and Effective Alternative in the Paediatric MRI Sedation

**DOI:** 10.7759/cureus.46787

**Published:** 2023-10-10

**Authors:** Kavya KG, Pooja N

**Affiliations:** 1 Department of Anaesthesiology, Rangadore Memorial Hospital, Bengaluru, IND; 2 Department of Anaesthesiology, Adichunchanagiri Institute of Medical Sciences, Nagamangala, IND

**Keywords:** intranasal dexmedetomidine, nonoperating room anaesthesia (nora), mri, paediatric, intravenous midazolam

## Abstract

Background and aims

MRI sedation in paediatrics includes challenges like respiratory depression, maintaining haemodynamic stability and use of neuroprotective drugs, since MRI is performed in remote places outside the operating room with a lack of support staff and nonavailability of choice of medications and equipments. The primary aim was to use a combination of the drugs to encounter the above challenges and look for its efficacy.

The secondary aim of the study was to determine the rate of successful completion of MRI in children using a combination of intranasal dexmedetomidine and intravenous midazolam - without the need for rescue sedatives.

Methods

This is an observational study involving 60 children in the age group between two months and six years undergoing an MRI. Children belonging to the American Society of Anesthesiology (ASA) 1 and 2 were given intranasal dexmedetomidine 3µg/kg, time to onset of sedation was noted and injection of midazolam 0.1 mg/kg was given intravenously. MRI was started once the child was asleep. Children who woke up during the MRI were supplemented with inj. propofol 0.5-1mg/kg and were documented.

Results

The median time duration for MRI was 38.7 min and the onset of sedation after intranasal dexmedetomidine was 18.7 min. The scan was successfully completed with a combination of intranasal dexmedetomidine and intravenous midazolam in 86.7% and only 13.3% of the children woke up either at the start or in between the scan and required the addition of propofol.

Conclusion

Drugs used for sedation during MRI should not cause respiratory depression and be safe for the developing brain. The above study has shown that a combination of intranasal dexmedetomidine and intravenous midazolam is effective and safe in performing MRIs in paediatrics.

## Introduction

Magnetic resonance imaging (MRI) use is growing exponentially in the pediatric population because of its associated lack of radiation, superior contrast resolution and excellent anatomic details. Children less than five years of age most commonly require moderate to deep sedation or an anaesthetic to ensure motionless conditions for MRI. MRI is one of the most common non-painful diagnostic modalities for which children require sedation [1].

MRI sedation is a challenging field in non-operative room anaesthesia (NORA). The challenges involved are avoiding respiratory depression, maintaining haemodynamic stability, the need for invasive ventilation and also use of neuroprotective drugs in the pediatric population. Our aim was to avoid invasive ventilation, and respiratory depression and ensure safety in children coming for MRI. Hence we decided to perform an MRI on the pediatric population using one such safe drug which is dexmedetomidine through the intranasal route. Previous studies have shown intranasal dexmedetomidine to be effective for MRI sedation, when used alone, only in infants [2]. Since this study included infants and children up to six years, we decided to add midazolam by intravenous (IV) route for better sedation and outcome. Dexmedetomidine is an agonist at the alpha-2 adreno receptors with sedative, anxiolytic and analgesic effects [3]. Intranasal administration is less invasive and has a significantly lower risk of respiratory depression and haemodynamic changes compared to IV administration [4].

The aim of the study is to determine the rate of successful completion of MRI imaging in children using a combination of intranasal dexmedetomidine and IV midazolam, without the need for rescue sedatives.

## Materials and methods

This is an observational study involving 60 children in the age group between two months to six years who came for MRI scans between January 2022 and January 2023. The study was approved by the Institutional Ethics Committee and registered with IEC/App/Jan/2022/008. Data analysis was carried out using the Statistical Package for the Social Sciences (PASW Statistics for Windows, Version 18.0, Chicago: SPSS Inc.).

**Figure 1:**
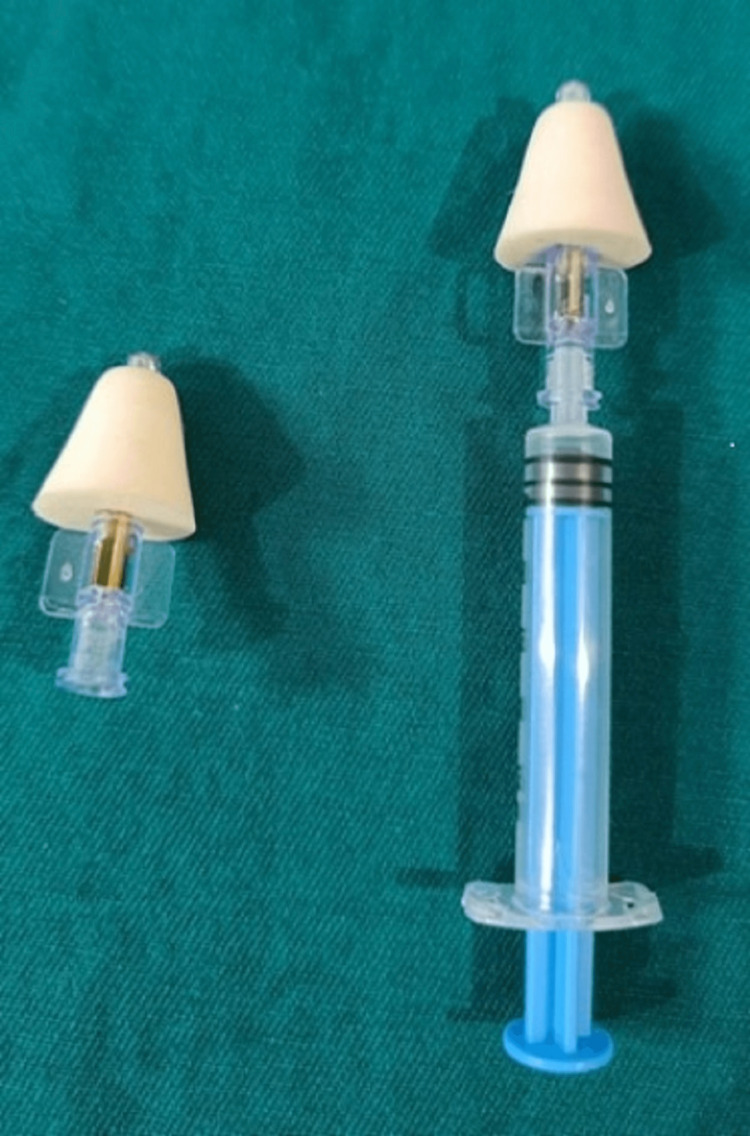
Nasal automizer to administer dexmedetomidine

All the children considered in the study belonged to the American Society of Anesthesiologists physical status classifications 1 and 2. After obtaining consent from the parents and initial assessment, children were given dexmedetomidine intranasally using nasal atomizers (Teleflex LMA MAD Nasal device, Morrisville, NC) as shown in Figure 1 in the dose of 3µg/kg.

A eutectic mixture of local anaesthetics like Prilox cream was applied to the best-visualized vein. Parents were advised to encourage kids to sleep. The time of onset of sedation after intranasal dexmedetomidine was noted and an IV cannula was placed. Injection midazolam was given in the dose of 0.1mg/kg intravenously. Children were taken into the MRI room, monitors attached, oxygen started via facemask, adequately wrapped to prevent hypothermia and the MRI scan was started. Airway equipments like I-gel (Intersurgical Ltd., UK), AMBU bags (Ambu A/S, Denmark), laryngoscopes and endotracheal tubes were kept ready in case of any airway emergencies. Pulse rate, respiratory rate, oxygen saturation and blood pressure were recorded every five minutes. Children who woke up during the MRI were supplemented with inj. propofol 0.5-1mg/kg and children who required supplementation of additional sedation were noted. The total duration of the MRI was noted. At the end of the MRI, all the kids were shifted to the recovery room and their vitals were recorded. Time for recovery was noted. Parents were advised to start orally once the child was fully awake and active.

## Results

The total number of study subjects were 60 out of which 35 were boys and 25 girls. The mean age and weight were 2.6 years and 15.4kg respectively. The median time duration of the MRI was 38.7 minutes. The median time for onset of sedation after intranasal dexmedetomidine was 18.7 minutes in this study.

MRI scan was successfully completed with the combination of intranasal dexmedetomidine and IV midazolam in 86.7% of the children, 13.3% of the children woke up either at the start of the MRI or in between and required an additional dose of propofol. A single sedative dose of propofol was sufficient to proceed with the MRI in these 13.3% of kids, neither a high dose nor was infusion of propofol required.

**Figure 2:**
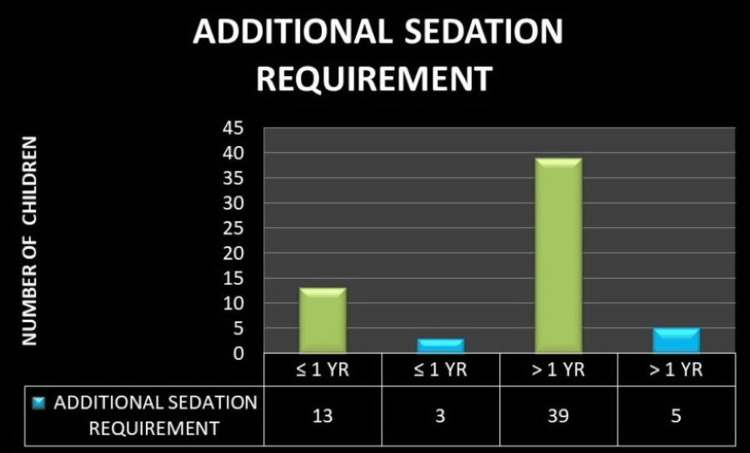
Graphical representation of study results The image shows the total number of children requiring additional sedation in blue and children who did not require additional sedation in green.

As shown in Figure 2, the total number of children less than one year was 16, out of which 13 did not require any additional sedation. Number of children more than one year was 44, out of which 39 children did not require any additional sedation. Therefore out of 60 children, 52 kids underwent MRI successfully with the above combination and only eight kids required additional sedation.

**Figure 3:**
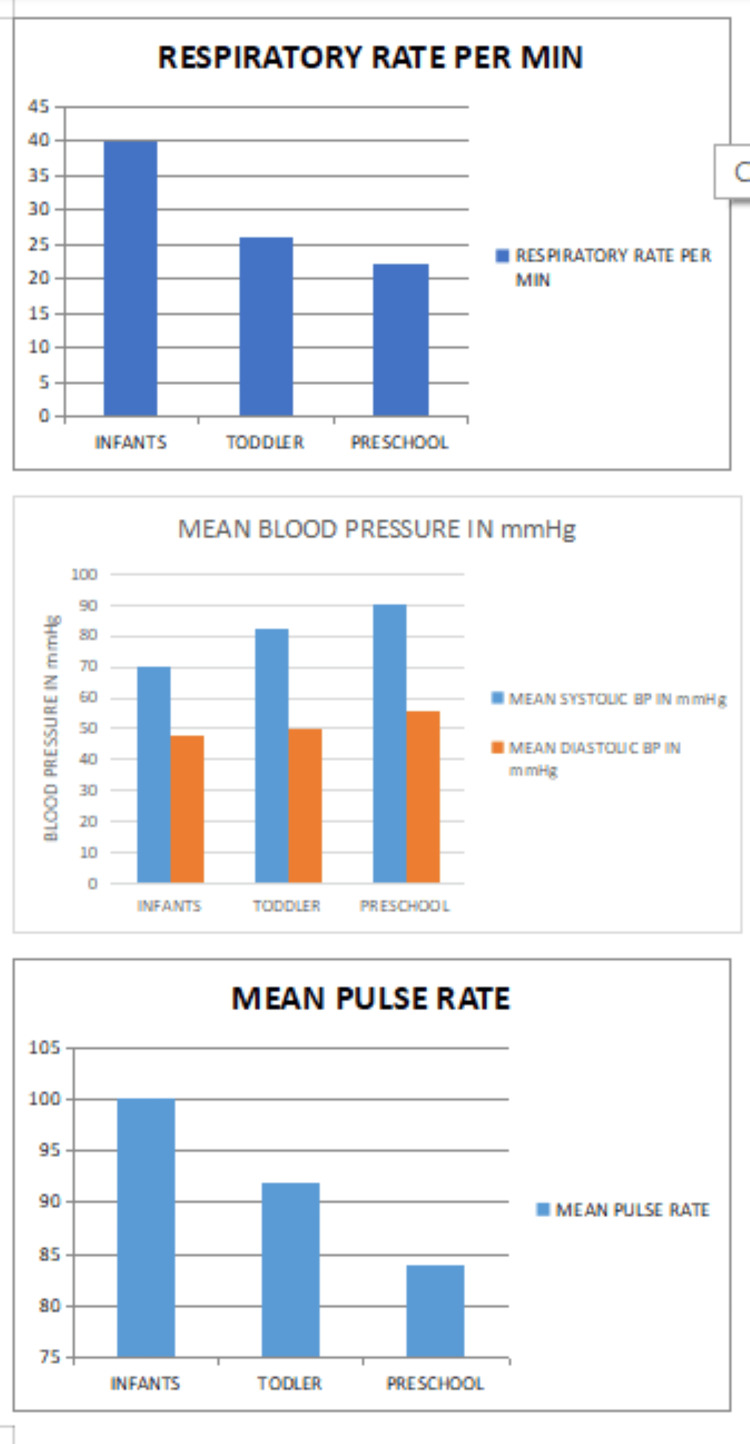
Graph showing mean respiratory rate, blood pressure and pulse rate of all the children during MRI. The respiratory rate, blood pressure and pulse rate were all maintained within normal limits for the age.

Pulse rate, blood pressure and saturation were maintained within normal limits for the age. No single case had respiratory depression or desaturation during the MRI. None of them had nausea and vomiting after the MRI scan (Figure 3).

## Discussion

Non-operating room anaesthesia (NORA) poses a challenge to the anaesthetists particularly in the pediatric population. The most common NORA in paediatrics is for performing MRI scans, where the children need to be immobile during the scans. Monitoring is a challenge in MRI suites because of the need to exclude ferromagnetic objects [1]. There may be a malfunction of equipment caused by changing magnetic fields. Therefore MRI-compatible equipment is required, like MRI-compatible monitors and infusion pumps which are very expensive.

Drugs used for sedation during MRI should not cause respiratory depression and be safe for the developing brain. Dexmedetomidine is one such drug with a favourable hemodynamic profile and relative ease of application, it does not cause respiratory depression and is also known to have neuroprotective properties [5]. Midazolam which is an anxiolytic and sedative anti-convulsant is a preferred drug in pediatric sedation because of its brief and consistent half-life and ideal for MRI sedation [6]. The present study is an observational study using the combination of these two drugs for MRI sedation and assessment of the effectiveness of the same.

Traditionally several sedatives like chloral hydrate, ketamine and propofol have been used for pediatric sedation during MRI. However, agents like chloral hydrate often fail to maintain the necessary depth of sedation to complete the procedure [7].

Oral chloral hydrate is found to have carcinogenic potential (although the risk of cancer from a single dose is inconclusive) [1]. Its neurotoxic properties have been investigated in an animal study. Preliminary results indicate that it causes neuroapoptosis in the cerebral cortex and the caudate-putamen complex in immature mouse pups in doses of 100 mg/kg or greater [8]. Chloral hydrate is also known for its other side effects like nausea, vomiting and agitation.

Ketamine is an N-methyl-D-aspartate (NMDA) receptor antagonist with sedative and analgesic properties. It is found to increase global and regional cerebral blood flow in healthy patients and, hence is a relative contraindication in children with significantly raised intracranial pressure, without controlled respiration [9]. Recovery from ketamine is notable because of the increased incidence of vomiting, hallucinations and agitation than other sedatives [10]. Even short-term use has been shown to have deleterious effects in some animal models. Ketamine is found to be associated with neuronal apoptosis after exposure in newborn animal model studies [11].

Propofol is a phenol derivative sedative, with profound respiratory depressant action. Respiratory complications have been reported in eight - 30% of the children [12]. When compared with propofol for sedation during MRI, dexmedetomidine provides adequate sedation during the scan but has a slower onset and recovery profile. When propofol is used alone for MRI, a high infusion dose is required which is associated with respiratory complications like upper airway obstruction and hypotension [13]. In children with suspected obstructive sleep apnea, the requirement of artificial airway support was significantly reduced when dexmedetomidine was used for MRI studies when compared with propofol for sedation. Mahmoud and colleagues (2009) have shown dexmedetomidine to be a useful anaesthetic agent for MRI evaluation of the airway in children with obstructive sleep apnea [14]. Considering the disadvantages of drugs like chloral hydrate, ketamine and high doses of propofol, a combination of dexmedetomidine and midazolam was used in the present study.

Benzodiazepines have anxiolytic, amnestic, sedative and anticonvulsant properties. Midazolam which is a short-acting benzodiazepine is the preferred drug for pediatric sedation because of its brief and more consistent half-life compared to diazepam [15,16]. The peak effect after IV administration of midazolam is 2-4 minutes with a duration of action of 45-60 minutes which is an ideal sedative for MRI that generally requires 35-45 minutes for completion. The intranasal route of midazolam administration causes irritation on the nasal mucosa, 85% of children who receive nasal midazolam will cry and complain of bitter aftertaste [17]. Because of this side effect, an IV route of midazolam was used in the present study.

Dexmedetomidine is a selective alpha-2 agonist which has seen a rapid increase in pediatric sedation over the past 10 years, because of a lack of respiratory depressant effects [18,19]. To date, dexmedetomidine does not cause neuroapoptosis (unlike all other sedatives). Dexmedetomidine may ameliorate developmental neurotoxicity associated with other sedatives and has neuroprotective properties [20].

Dexmedetomidine use in neonates and children has expanded to include the prevention of emergence delirium, postoperative pain management, invasive and non-invasive procedural sedation and the management of opioid withdrawal [21].

Dexmedetomidine can be administered by IV, intramuscular, buccal, oral and intranasal routes. Sedation and anxiolysis are mediated through action on alpha-2 receptors on locus coeruleus. The suppression of locus coeruleus neurons is similar to normal sleep. It provides respiratory stability with no ventilatory depression. Analgesia is mediated primarily via the spinal cord, although there is evidence that supraspinal and peripheral nerves may contribute to this effect [22]. Dexmedetomidine via the intranasal route has been found to be effective for brainstem evoked response audiometry (BERA) and electroencephalogram (EEG) examinations in paediatrics [23]. Sulton et al. demonstrated that a dose of 3µg/kg of intranasal dexmedetomidine in combination with intranasal midazolam was effective for MRI of children. All 224 procedures were completed and there were no adverse effects [24]. However since intranasal midazolam is an irritant to the nasal mucosa and has a bitter aftertaste, the IV route was used in the present study.

Intranasal dexmedetomidine used as premedication in pediatric patients with dosages of 2-3µg/kg has shown the onset of action to be 10-33 min [25]. Studies have shown that intranasal dexmedetomidine administration provides some flexibility as long as it is given at least 30, and preferably 45min, in advance [26]. Intranasal dexmedetomidine peak concentration is achieved in 37 minutes and maximal sedative effect is seen in 45 minutes when given at the dose of 2-3µg/kg. The concentration time profiles indicated that the plasma concentration decreased quite rapidly and higher initial doses or repeated doses may be needed for clinical efficacy for longer procedures. Moreover, sedation with dexmedetomidine maintains patients' responsiveness and arousability. This may be challenging in procedures where patients are expected to stay immobile. Intranasal dexmedetomidine as a sole agent might not be sufficient for procedural MRI sedation of pediatric patients and combination with other sedative agents may be needed [27]. Hence we decided to combine another sedative agent and considering the safety profile, a combination of IV midazolam and intranasal dexmedetomidine was used and the effectiveness of this combination in completion of MRI was assessed. Out of the total 60 cases in the study, 52 MRI scans were successfully completed with this combination without the requirement of additional sedatives. Only eight children required the addition of injection propofol during the MRI scan as a single bolus injection in the dose of 0.5-1mg/kg.

General anaesthesia with tracheal intubation or supra-glottic airway devices and mechanical ventilation is too invasive and resource-consuming. In developing countries, intranasal dexmedetomidine and IV midazolam combination also minimizes the need to use expensive MRI-compatible syringe pumps and long infusion sets. There was no single case of respiratory depression or haemodynamic instability in the present study. The combination of sedation used in the study has shown promising results in the uncooperative pediatric population coming for MRI scans.

The limitation of the present study is that there are only 16 kids under one year of age; more studies are required in this particular age group. It is a single-centric study, multicentric studies are essential in infants and children.

## Conclusions

Drugs used for sedation during MRI should not cause respiratory depression and be safe for the developing brain. Dexmedetomidine administered via the intranasal route has a favourable haemodynamic profile and relative ease of application, it does not cause respiratory depression and is also known to have neuroprotective properties. Midazolam which is an anxiolytic and sedative anticonvulsant is a preferred drug in pediatric sedation because of its brief and consistent half-life and ideal for MRI sedations. The above study has shown that a combination of intranasal dexmedetomidine and IV midazolam is effective and safe in performing MRIs in paediatrics.
